# Combined Immune Checkpoint Inhibitors and Radiation Therapy in Patients with Multiple Myeloma and Extramedullary Medullary Disease: A Real-World Retrospective Analysis

**DOI:** 10.3390/cancers18121996

**Published:** 2026-06-19

**Authors:** Lili Zhang, Ayrton Bangolo, Behzad Amoozgar, Sarvarinder Gill, Jiahe Zhao, Gurpavitar Singh Bhullar, Sindhu Singareddy, Shubhangi Singh, Henry Ortiz, Alicia Muench, Sarah Peake, Komal Azam, Winnie Noe, Jericho Ghanem, Eme De Graaf, Ashrika Sookoo, Manjunath N. R. K. Reddy, Selbin Boban, Sikder Sakil, Duval Samwaru, Keerthi Sadasivan, Julia Baracewicz, Sai Manoja Bheemineni, Sahejdeep Chohan, Simcha Weissman, Harsh Parmar, Pooja Phull, David Siegel, David H. Vesole, Noa Biran

**Affiliations:** 1 Department of Hematology and Oncology, John Theurer Cancer Center, Hackensack, NJ 07601, USA; lili.zhang@hmhn.org (L.Z.); behzad.amoozgar@hmhn.org (B.A.); sarvarinder.gill@hnmhn.org (S.G.); jiahe.zhao@hmhn.org (J.Z.); 2 Department of Medicine, Palisades Medical Center, North Bergen, NJ 07047, USA; gurpavitar.singhbhullar@hmhn.org (G.S.B.); sindhu.singareddy@hmhn.org (S.S.); shubhangi.singh@hmhn.org (S.S.); henry.ortiz@hmhn.org (H.O.); alicia.muench@hmhn.org (A.M.); sarah.peake@hmhn.org (S.P.); komal.azam@hmhn.org (K.A.); winnie.noe@hmhn.org (W.N.); jericho.ghanem@hmhn.org (J.G.); eme.degraaf@hmhn.org (E.D.G.); ashrika.sookoo@hmhn.org (A.S.); manjunath.n.r.k.reddy@hmhn.org (M.N.R.K.R.); selbin.boban@hmhn.org (S.B.); sikder.sakil@hmhn.org (S.S.); duval.samwaru@hmhn.org (D.S.); keerthi.sadasivan@hmhn.org (K.S.); julia.baracewicz@hmhn.org (J.B.); sai.manoja.bheemineni@hmhn.org (S.M.B.); sahejdeep.chohan@hmhn.org (S.C.); simcha.weissman@hmhn.org (S.W.); 3 Division of Myeloma, John Theurer Cancer Center, Hackensack, NJ 07601, USA; harsh.parmar63@hmhn.org (H.P.); pooja.phull54@hmhn.org (P.P.); david.ssiege89l@hmhn.org (D.S.); david.h.vesole@hmhn.org (D.H.V.); noa.birann67@hmhn.org (N.B.)

**Keywords:** multiple myeloma (MM), extramedullary disease (EMD), immune checkpoint inhibitors (ICI), radiation therapy (RT), immunotherapy, nivolumab, pembrolizumab, refractory myeloma, abscopal effect, CAR-T therapy

## Abstract

**Background/Objectives:** Extramedullary disease (EMD) is an aggressive and treatment-resistant manifestation of multiple myeloma with limited therapeutic options, particularly in heavily pretreated patients. **Methods:** We conducted a retrospective study to evaluate the efficacy and safety of concurrent immune checkpoint inhibitors (ICIs) and radiation therapy (RT) in patients with EMD treated at Hackensack University Medical Center and John Theurer Cancer Center between January 2016 and May 2025. Patients were included if they had confirmed EMD and received nivolumab or pembrolizumab with concurrent RT. **Results:** A total of 21 patients were included, representing a high-risk cohort with a median of 6 prior lines of therapy (range 2–13), including 47.6% triple-class refractory and 19.0% penta-refractory disease. The overall response rate (ORR) was 47.6%, with a clinical benefit rate of 57.1%. Despite these responses, median progression-free survival (PFS) and overall survival (OS) were 4 and 12 months, respectively. Notably, two patients achieved complete responses with nivolumab and RT early in their treatment course following cellular therapy and remain disease-free at last follow-up. The combination of ICIs and RT was generally well-tolerated, with manageable immune-related adverse events and no treatment-related deaths. **Conclusions:** These findings suggest that concurrent ICI and RT may provide a signal of treatment responses in a subset of patients with advanced EMD, although durability remains limited. Further prospective studies are warranted to further define the role of this combination and identify patients most likely to benefit.

## Simple Summary

Patients with multiple myeloma who have extramedullary disease (EMD) represent a high-risk population with limited treatment options and poor outcomes, which represent a high unmet need. EMD is defined as organ involvement or soft-tissue plasmacytomas that are not contiguous with bone lesions; paraskeletal plasmacytomas contiguous with bone are excluded. EMD is an aggressive and difficult-to-treat manifestation of myeloma, especially in patients who have received multiple prior lines of therapy. In this study, we evaluated the use of immune checkpoint inhibitors (ICIs; nivolumab or pembrolizumab) combined with radiation therapy (RT) in 21 heavily pretreated EMD myeloma patients. The combination of ICIs and RT led to meaningful responses in some patients, with an overall response rate of 47.6%, although most responses were not durable. Notably, two patients achieved complete responses and remained disease-free at last follow-up, raising the hypothesis that certain patients, particularly those treated earlier in their disease course or after cellular therapies, may derive greater benefit. Treatment was generally well-tolerated, with manageable adverse events and no treatment-related deaths. These findings suggest that combining immunotherapy with radiation may be a feasible and potentially effective option for selected patients with advanced EMD. Further prospective studies are needed to confirm these results and identify patients most likely to benefit.

## 1. Introduction

Multiple myeloma (MM) is a plasma cell malignancy characterized by clonal proliferation within the bone marrow; however, a subset of patients develop extramedullary disease (EMD), defined by plasma cell infiltration outside the bone marrow microenvironment. EMD represents a distinct and aggressive clinical entity associated with adverse outcomes, including inferior progression-free survival (PFS) and overall survival (OS), even in the era of novel agents and cellular therapies [1,2]. The biology of EMD is complex and involves loss of adhesion molecules, increased genomic instability, and immune evasion, which contribute to its resistance to conventional therapies and propensity for dissemination [3].

The incidence of EMD appears to be increasing, partly due to improved imaging modalities and longer survival of MM patients, allowing for clonal evolution over time [4]. Importantly, EMD is frequently associated with high-risk cytogenetic abnormalities, such as del(17p), t(4;14), and t(14;16), further contributing to its poor prognosis [5]. Patients with EMD often present with soft-tissue plasmacytomas or visceral organ involvement, both of which are associated with more aggressive disease behavior and limited therapeutic options [6].

While CAR-T cell therapies and bispecific antibodies have expanded the therapeutic landscape, EMD continues to pose a significant clinical challenge, particularly in the relapsed/refractory setting. The CARTITUTE-1 trial reported ORR of 95–100% across all subgroups including patients with plasmacytomas; however, the EMD cohort was small (~20 patients with true EMD), the vast majority were BCMA-naive (>88%), and the duration of response, PFS, and OS were significantly shorter compared to patients without EMD [7]. In the CARTITUTE-4 trial, the EMD subgroup analysis demonstrated a median PFS of 13 months versus 4 months for cilta-cel versus standard of care (HR 0.71; 95% CI, 0.34–1.49) [8], suggesting a trend toward benefit that did not reach statistical significance in this limited subgroup. A meta-analysis of 42 studies pooling 242 EMD patients treated with BCMA-directed CAR-T (including cilta-cel and ide-cel) found a pooled ORR of 79%, which was substantially lower than the ORR of 90% in the non-EMD patients [9]. Response rates among patients with EMD treated with bispecific antibodies are generally lower. Talquetamab monotherapy achieved an ORR of 40–45%, with a CR rate of only 15% [10]; Teclistamab monotherapy has an ORR of 36% in a retrospective cohort, with CR of ~19%; Elranatamab was reported to have an ORR of 39% in EMD patients [11]; linvoseltamab showed dose-dependent activity, with ≥ PR in 26.7% at 50 mg and 56.3% at 200 mg [12]. Notably, the combination of talquetamab plus teclistamab has demonstrated substantially improved outcomes in true EMD in the Phase 2 RedirectTT-1 study, with an ORR of 79% (95% CI, 69–87), CR or better in 54%, and median PFS of 15.4 months—results that compare favorably to less than 3 months with standard therapies and less than 6 months with bispecific monotherapies in this population [13]. Overall, CAR-T cell therapies demonstrate higher response rates in EMD compared to bispecific antibody monotherapy. However, even among patients who initially respond to CAR-T, outcomes remain challenging. Post-CAR-T relapse in EMD patients carries a particularly poor prognosis, with a median OS of only 4.8 months from progression compared to 21 months in non-EMD patients [14]. These findings underscore that while CAR-T and bispecific therapies represent meaningful advances for EMD, durable disease control remains an unmet need, and novel strategies warrant further investigation in this high-risk population.

Immune checkpoint inhibitors (ICIs), including programmed death-1 (PD-1) inhibitors such as nivolumab and pembrolizumab, have revolutionized the treatment of several malignancies by restoring antitumor immune responses. Despite the broad efficacy of ICIs across most malignancies, their efficacy in patients with multiple myeloma has not been consistently proven [15,16]. Early-phase studies suggested potential activity, particularly in combination with IMiDs, but subsequent phase III trials (KEYNOTE-183 and KEYNOTE-185) demonstrated increased toxicity and mortality with pembrolizumab-based combinations, leading to early termination of these studies and regulatory warnings [17]. In patients with EMD, RT is commonly used for bulky or symptomatic plasmacytomas and has demonstrated high local control rates [18]. Beyond its local effects, RT may also have immunomodulatory properties, including enhanced antigen presentation, increased tumor infiltration by immune cells, and potential induction of systemic antitumor responses (the “abscopal effect”) [18]. These properties provide a strong rationale for combining RT with ICIs to potentially enhance systemic immune responses in MM.

Given the limited treatment options and poor outcomes associated with EMD, particularly in heavily pretreated and refractory patients, there is a critical need to explore novel therapeutic approaches. The combination of ICIs with RT represents a biologically plausible strategy that may help overcome some of the immune resistance mechanisms in EMD. In this study, we conducted a retrospective analysis of patients with EMD treated with concurrent PD-1 blockade (nivolumab or pembrolizumab) and radiation therapy at our institution, with the aim of evaluating the efficacy, safety, and potential clinical benefit of this combination in a real-world, high-risk population.

## 2. Methods

### 2.1. Study Design and Patient Selection

Following Institutional Review Board (IRB) approval, a retrospective chart review was conducted of patients with extramedullary multiple myeloma treated at Hackensack University Medical Center and John Theurer Cancer Center between 1 January 2016, and 31 December 2024. Patients were included if they (1) had confirmed diagnosis of EMD that were detected through imagining including CT, PET/CT or MRI, and maybe confirmed by biopsy; (2) received treatment with either nivolumab or pembrolizumab; (3) received concurrent radiation therapy within 4 weeks of initiation of ICIs; and (4) had adequate medical records for analysis.

### 2.2. Treatment Protocols

Immune checkpoint inhibitors were administered according to standard dosing regimens: nivolumab at 240 mg every 2 weeks or 480 mg every 4 weeks, and pembrolizumab at 200 mg every 3 weeks. Radiation therapy was delivered using external beam radiotherapy (EBRT) techniques, with total doses ranging from 2000 to 3000 cGy given in 10–15 fractions, depending on lesion size, location, and clinical intent. Concurrent therapy was defined as radiation administration within ±4 weeks of ICI dosing.

Additional antimyeloma therapies, including immunomodulatory drugs (lenalidomide, pomalidomide, or thalidomide), were administered at the discretion of the treating physician based on disease characteristics and prior treatment history.

### 2.3. Data Collection and Statistical Analysis

Clinical data extracted included patient demographics, disease characteristics, prior treatment history, response to therapy, survival outcomes, and toxicity information. Response was assessed according to International Myeloma Working Group (IMWG) criteria, with the addition of minimal response (MR) as previously defined. Extramedullary responses were evaluated using serologic markers (M-protein, free light chains) and radiographic imaging (CT, PET/CT, or MRI) in patients with secretory disease, whereas radiographic imaging alone was used in patients with nonsecretory disease. Serologic markers were assessed monthly, while imaging was performed approximately every 3 months or earlier if clinically indicated by suspicion of disease progression.

Progression-free survival was calculated from the start of the day of starting ICIs to disease progression or death from any cause, whichever occurs first. Overall survival was calculated from the day of starting ICIs to death from any cause.

Given the exploratory nature of this study and the limited sample size, statistical analyses were primarily descriptive. Categorical variables were summarized using frequencies and percentages, while continuous variables were summarized using medians and ranges. Survival outcomes were estimated using the Kaplan–Meier method. No formal comparative or multivariable analyses were performed because the sample size was insufficient to support reliable statistical inference. All analyses were conducted using SPSS version 27.0 (IBM Corp., Armonk, NY, USA).

## 3. Results

### 3.1. Baseline Characteristics

Twenty-one patients with EMD who received concurrent immune ICIs (nivolumab or pembrolizumab) and radiation therapy were included in the analysis. The majority were male (66.7%) and aged ≥40 years (85.8%), with a predominance of Non-Hispanic White patients (66.7%). Disease burden was substantial, with patients distributed across ISS stages, including 28.6% each in stages I and III, and 23.8% with unknown staging. Nearly half of the cohort (42.9%) harbored high-risk cytogenetic features. The most common disease subtypes were IgG (47.6%) and IgA (28.6%), with kappa light chain predominance (52.4%) as seen in Table 1.

The median number of prior lines of therapy was six (range, 2–13). Ten patients (47.6%) were triple-class refractory, and four (19.0%) were penta-refractory. All patients had true EMD, including six patients (28.6%) with visceral organ involvement. All patients (100.0%) had previously undergone autologous stem cell transplantation (ASCT), five (23.8%) had received prior CAR-T cell therapy, and five (23.8%) had received both ASCT and CAR-T therapy.

### 3.2. Treatment

Ten patients (47.6%) received nivolumab in combination with RT, while 11 patients (52.4%) received pembrolizumab with RT. Radiation was delivered as EBRT directed to EMD. Most patients received a total dose of 3000 cGy administered in 10–15 fractions. Radiation treatment fields were individualized according to the location and extent of extramedullary disease, including soft-tissue and visceral lesions.

Treatment regimens were heterogeneous. In addition to ICI and RT, ten patients (47.6%) received concomitant pomalidomide, four patients (19.0%) received lenalidomide, and one patient (4.8%) received thalidomide. Six patients (28.6%) received ICI with RT without concomitant immunomodulatory drugs.

### 3.3. Efficacy Results

With a median follow-up of 9 months (range 1–96), the ORR (CR + PR) was 47.6%, including two complete responses (9.5%) and eight partial responses (38.1%), with an additional two patients achieving minimal response (9.5%), resulting in a clinical benefit rate of 57.1% as seen in Table 2. Stable disease was observed in three patients (14.3%). Four patients experienced progressive disease (19%), and two patients had unknown responses (9.5%) as seen in Table 2.

**Figure 1:**
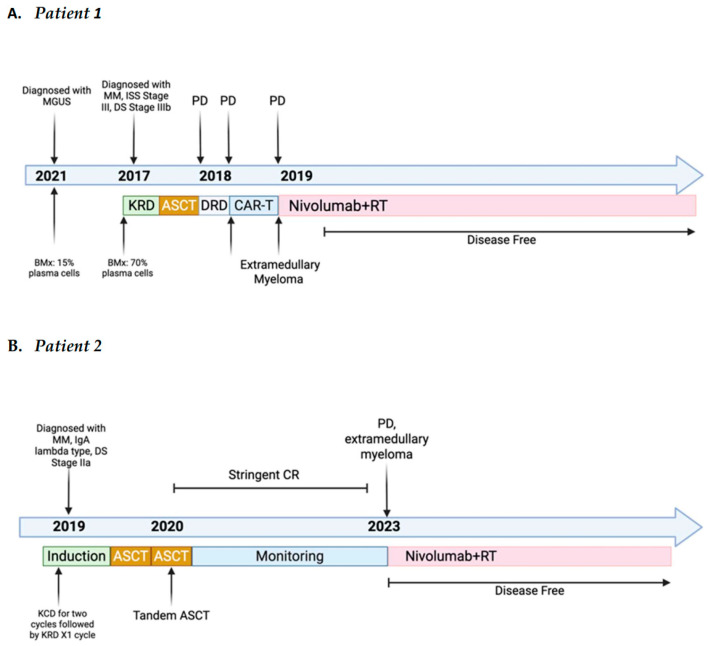
Treatment history and timeline of patients achieving complete response after receiving nivolumab and RT. (**A**) Patient 1: The patient was diagnosed with MGUS in 2011. Bone marrow biopsy showed 15% of plasma cells. Patient then progressed to symptomatic MM in 2017, with 70% bone marrow plasma cell involvement and ISS Stage III/DS Stage IIIb disease. Patient received induction therapy with KCD followed by ASCT. Disease progressed in 2018, which prompted treatment with DRD, followed by CAR-T cell therapy for subsequent relapse. The patient later developed EMD and experienced additional disease progression in 2019 and was treated with Nivo/RT, which resulted in a complete and durable remission. The patient has remained disease free during long-term follow-up. (**B**) Patient 2: The patient was diagnosed with IgA λ MM in 2019, DS Stage IIa disease. Initial therapy consisted of two cycles of KCD followed by one cycle of KRD induction therapy and tandem ASCT. The patient subsequently achieved an sCR and remained under surveillance until 2023. Disease progression occurred with the development of EMD. Nivo/RT was started and resulted in complete disease remission. Patient has remained disease free throughout follow-up after treatment initiation. ASCT, autologous stem cell transplantation; BMx, bone marrow biopsy; CAR-T, chimeric antigen receptor T-cell therapy; CR, complete response; DRD, daratumumab, lenalidomide, and dexamethasone; DS, Durie–Salmon staging system; ISS, International Staging System; KCD, carfilzomib, cyclophosphamide, and dexamethasone; KRD, carfilzomib, lenalidomide, and dexamethasone; MGUS, monoclonal gammopathy of undetermined significance; MM, multiple myeloma; PD, progressive disease; RT, radiotherapy.

Detailed treatment details and timeline are described in Figure 1.

**Figure 2:**
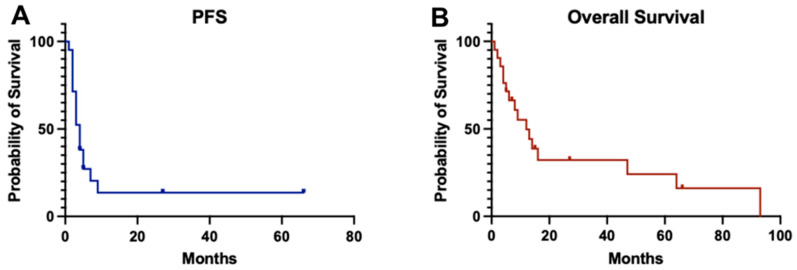
Survival outcome for patients with EMD under treatment with ICI and RT. Kaplan–Meier estimate of (**A**) PFS and (**B**) OS. PFS was defined as the initiation of ICI to disease progression or death from any cause, whichever occurs first. OS was defined as the time from initiation of ICI to death from any cause. Tick marks indicate censored observations. Median PFS was 4 months. Median OS is 12 months. EMD, extramedullary disease; ICI, immune checkpoint inhibitor; OS, overall survival; PFS, progression-free survival; RT, radiotherapy.

Median progression-free survival was 4 months, and median overall survival was 12 months (Figure 2).

Notably, the two patients who achieved complete responses both received nivolumab with concurrent radiation therapy. These patients had received treatment shortly after cellular therapy: one following ASCT as second-line therapy and the other following idecabtagene vicleucel CAR-T therapy as third-line treatment. Both patients remained in complete remission without evidence of disease progression at the time of last follow-up.

### 3.4. Safety Results

The combination therapy was generally well-tolerated. Treatment-related adverse events included grade 3 pancreatitis in one patient (4.8%), grade 1 skin toxicity in two patients (9.5%), subclinical hypothyroidism in two patients (9.5%), and hemophagocytic lymphohistiocytosis (HLH) in one patient (4.8%).

One patient developed CTCAE grade 3 immune-related pancreatitis approximately 6 weeks after completion of immune checkpoint inhibitor (ICI) therapy. The patient presented with abdominal pain and a serum lipase level exceeding three times the upper limit of normal. The event was considered treatment-related based on its clinical presentation and temporal association with ICI exposure. The patient was treated with prednisone at 1 mg/kg daily for 4 days followed by a gradual steroid taper, resulting in complete resolution of symptoms and normalization of laboratory abnormalities.

One patient developed HLH shortly after completion of ICI therapy. Clinical manifestations included fever, splenomegaly, pancytopenia, elevated liver function tests, hypertriglyceridemia (>300 mg/dL), and marked hyperferritinemia (ferritin > 10,000 ng/mL). Based on the timing of onset and clinical findings, the event was considered immune-related. The patient was treated with anakinra in combination with prednisone followed by a prolonged steroid taper, leading to clinical improvement and resolution of HLH-related manifestations.

Both serious immune-related adverse events resolved with appropriate medical management. No treatment-related deaths were observed.

## 4. Discussion

This retrospective study of 21 patients with EMD evaluated the use of concurrent immune checkpoint inhibitors (nivolumab or pembrolizumab) and radiation therapy in a heavily pretreated, high-risk population characterized by a median of 6 prior lines of therapy, frequent triple- (47.6%) and penta-refractory disease (19.0%), and substantial prior exposure to cellular therapies. Most patients received 3000 cGy of radiation, often in combination with IMiDs. The overall response rate was 47.6%, with a clinical benefit rate of 57.1%, although responses were generally not durable, with a median progression-free survival of 4 months and overall survival of 12 months. Notably, two patients achieved complete responses with nivolumab plus radiation early in their treatment course following cellular therapy and remain disease-free, generating hypotheses for timing and immune priming. The combination was overall well-tolerated, with manageable immune-related adverse events and no treatment-related deaths, supporting the feasibility of this approach in a difficult-to-treat population.

These baseline characteristics are consistent with the current literature describing EMD as an aggressive, biologically high-risk phenotype. Prior studies have shown that EMD is associated with inferior progression-free and overall survival and is over-represented among genomically high-risk myeloma, even in the era of novel therapies [5]. In this context, the finding that 42.9% of patients in our cohort had high-risk FISH is highly relevant and closely aligns with published data; a systematic review reported high-risk cytogenetics in approximately 41% of patients with EMD, particularly in true extramedullary disease [19]. The predominance of IgG disease followed by IgA disease is also consistent with reported EMD cohorts, although the presence of extramedullary involvement itself likely carries greater significance than immunoglobulin subtype.

The inclusion of patients treated with nivolumab or pembrolizumab with concurrent radiation is notable because checkpoint inhibition has shown limited and inconsistent success in multiple myeloma, particularly in monotherapy settings, despite strong biologic rationale related to immune exhaustion and PD-1/PD-L1 signaling pathways [20,21,22]. Prior studies have demonstrated that RT remains an important modality for palliation, symptom control, and achieving high local response rates in localized MM lesions [18,23]. While these data are derived from localized disease rather than extramedullary disease (EMD), similar principles are generally considered applicable to EMD, which often presents as localized soft-tissue or organ-based lesions amenable to local control with RT.

The high-risk features observed in this cohort including male predominance, high-risk cytogenetics, advanced disease stage, and aggressive extramedullary presentation, support the rationale for evaluating whether radiation can augment antitumor immunity and potentially improve the activity of checkpoint blockade in a population with otherwise limited therapeutic options.

The median of six prior lines of therapy, along with 47.6% triple-class refractory and 19.0% penta-refractory disease, underscores the advanced nature of this cohort, for whom conventional options are limited. Prior exposure to cellular therapies was also common. EMD has been associated with inferior outcomes even after BCMA-directed CAR-T therapy, and post-CAR-T relapse with extramedullary involvement remains an area of unmet need [24,25,26,27,28]. The predominance of soft-tissue disease is clinically relevant because localized RT remains commonly used for symptomatic, bulky, or anatomically threatening plasmacytomas, while visceral organ involvement generally reflects even more aggressive disease biology.

The therapeutic approach in this cohort combined local RT, PD-1 blockade, and in many cases IMiD therapy. Most patients received 3000 cGy in 10–15 fractions, which is consistent with commonly used palliative myeloma RT schedules; ILROG consensus guidelines support fractionated regimen such as 30 Gy in 10 fractions as a reasonable option in selected cases [29].

The use of pembrolizumab or nivolumab is biologically plausible in combination with radiation, which may promote antigen release and immune priming. However, prior phase III trials in multiple myeloma evaluating PD-1 inhibitors in combination with IMiD demonstrated increased toxicity and excess mortality in KEYNOTE-183 and KEYNOTE-185 trials, leading to early termination and regulatory caution regarding these combinations outside clinical trials [17]. In this context, our cohort reflects a real-world salvage strategy in a population largely excluded from standard trials, but the high proportion receiving concurrent pomalidomide or lenalidomide means safety and efficacy signals should be interpreted cautiously and ideally validated prospectively.

Our observed response rate appears favorable compared with historical outcomes in heavily pretreated EMD, where responses are often limited and short-lived. In a pooled analysis of triple-class exposed RRMM with EMD, the ORR of approximately 24.1%, median PFS of 2.66 months, and median OS of 7.16 months have been reported, highlighting the poor prognosis of this subgroup [27]. In that context, the ORR of 47.6% and clinical benefit rate of 57.1% observed in our cohort suggest potential activity of this combined approach in selected patients. However, the median PFS of 4 months and OS of 12 months remain modest, indicating that despite initial responses, disease control is often not durable, consistent with the aggressive biology and treatment resistance associated with EMD.

Checkpoint inhibitor studies in myeloma have been mixed: early-phase data suggested potential activity, including anecdotal reports of responses in combination with RT, but pembrolizumab combinations with IMiDs later showed an unfavorable benefit–risk profile in KEYNOTE-183, leading to FDA action and caution against PD-1/PD-L1 blockade with thalidomide analogs outside controlled trials [30]. In our study, the two complete responses observed in patients receiving nivolumab plus radiation are insufficient to draw definitive conclusions but raise hypotheses regarding potential determinants of response, including timing of therapy, disease burden, prior immune priming from transplant or CAR-T therapy, and radiation-mediated immune activation. These observations are speculative and require prospective validation in appropriately controlled studies.

The safety profile observed in this cohort appears acceptable and consistent with expected immune-related adverse events from PD-1 blockade. Immune-related adverse events, including hypothyroidism, skin toxicity, pancreatitis, and HLH, were manageable with standard immunosuppressive approaches, including corticosteroids and supportive therapy. Importantly, no treatment-related deaths were observed, which is reassuring given historical concerns regarding checkpoint inhibitor combinations in multiple myeloma, particularly in combination with IMiDs. The addition of radiation did not appear to result in an unexpected increase in severe toxicity, consistent with prior data suggesting that radiotherapy administered concurrently with immune checkpoint inhibitors is not associated with a significant increase in high-grade adverse events across tumor types [31].

This study has several notable strengths, particularly given the rarity and clinical complexity of extramedullary disease. It evaluates a real-world cohort of high-risk, heavily pretreated patients, many of whom had triple-class or penta-refractory disease and prior exposure to cellular therapies, reflecting a population frequently underrepresented in prospective clinical trials. In addition, this study explores the combination of immune checkpoint inhibition and radiation therapy, a biologically plausible but relatively under-investigated treatment strategy in multiple myeloma.

However, several limitations warrant consideration. The retrospective design, small sample size, absence of a comparator group, and heterogeneity in concurrent therapies limit the ability to draw definitive conclusions regarding efficacy or causality. Response assessment was subject to the inherent limitations of retrospective data collection, including non-standardized imaging schedules and the absence of central radiographic review. Additionally, the high proportion of patients receiving concurrent immunomodulatory agents may have influenced both efficacy and toxicity outcomes. Therefore, the findings should be considered exploratory and hypothesis-generating.

Despite these limitations, the observed response rate and the occurrence of durable complete remission in two patients suggest that immune checkpoint blockade combined with radiation therapy may have activity in a subset of patients with EMD. Future prospective studies incorporating biomarker analyses and more uniform treatment approaches will be important to clarify the contribution of immune checkpoint inhibition, define optimal patient selection, and determine whether earlier use of this strategy can improve long-term outcomes.

## 5. Conclusions

In this retrospective study, analysis of patients with extramedullary multiple myeloma, concurrent immune checkpoint inhibitor therapy and radiation therapy demonstrated clinically meaningful activity, with an overall response rate of 47.6% and an acceptable safety profile. Although most responses were not durable, two patients achieved sustained complete remissions, suggesting that selected patients may derive substantial benefit from this approach. These findings support further prospective investigation of immune checkpoint inhibition combined with radiation therapy in EMD, particularly in earlier disease settings and following cellular therapies where immune priming may enhance treatment efficacy.

## Figures and Tables

**Table 1:** Patient baseline Characteristics (*N* = 21).

Characteristic	Number	Percentage
Gender		
Male	14	66.7%
Female	7	33.3%
Age at Diagnosis (Years)		
0–39	3	14.3
40–59	9	42.9%
60–79	9	42.9%
80+	0	0
Race		
Non-Hispanic White	14	66.7%
Non-Hispanic Black	3	14.3%
Hispanic	3	14.3
Others	1	4.8%
ISS Stage		
Stage I	6	28.6%
Stage II	4	19.0%
Stage III	6	28.6%
Unknown	5	23.8%
Disease Subtype		
IgG	10	47.6%
IgA	6	29.6%
Light Chain	4	19.0%
Others	1	4.8%
Light Chain Subtype		
Kappa	11	52.4%
Lambda	9	42.9%
Others	1	4.8%
High-Risk Fish		
Yes	9	42.9%
No	12	57.1%
Prior lines of therapy (median, range)	6 (2–13)	
Refractoriness		
Triple-class refractory	10	47.6%
Penta-refractory	4	19.0%
Extramedullary sites		
Soft-tissue masses	15	71.4%
Organ involvement	6	28.6%
Cellular immunotherapy prior to ICI + RT		
Autologous transplant	21	100.0%
CAR-T therapy	5	23.8%
Both	5	23.8%

**Table 2:** Treatment Outcomes (N = 21).

Response Category	Number	Percentage
Complete Response (CR)	2	9.5%
Partial Response (PR)	8	38.1%
Minimal Response (MR)	2	9.5%
Stable Disease (SD)	3	14.3%
Progressive Disease (PD)	4	19.0%
Unknown	2	9.5%
Overall Response Rate (CR + PR)	10	47.6%
Clinical Benefit Rate (CR + PR + MR)	12	57.1%
Median PFS	4 (months)	
Median OS	12 (months)	

## Data Availability

The complete datasets used and/or analyzed during this study are available from the corresponding author upon request. Requests can be made through the corresponding author (Ayrton Bangolo; Email: Ayrton.bangolo@hmhn.org).

## List of Abbreviations

**ASCT**	Autologous Stem Cell Transplant
**CAR-T**	Chimeric Antigen Receptor T-cell
**CR**	Complete Response
**CT**	Computed Tomography
**DRD**	Daratumumab, Revlimid, and Dexamethasone
**EMD**	Extramedullary Disease
**EBRT**	External beam radiotherapy
**FISH**	Fluorescence In Situ Hybridization
**HLH**	Hemophagocytic Lymphohistiocytosis
**ICIs**	Immune Checkpoint Inhibitors
**ILROG**	International Lymphoma Radiation Oncology Group
**IMiDs**	Immunomodulatory Drugs
**irAE**	Immune-Related Adverse Events
**IRB**	Institutional Review Board
**KCD**	Carfilzomib, Cyclophosphamide and Dexamethasone
**KRD**	Carfilzomib, Revlimid and Dexamethasone
**MM**	Multiple Myeloma
**MR**	Minimal Response
**MRI**	Magnetic Resonance Imaging
**ORR**	Overall Response Rate
**OS**	Overall Survival
**PD**	Progression of Disease
**PD-1**	Programmed Death-1
**PET/CT**	Positron Emission Tomography/Computed Tomography
**PFS**	Progression-Free Survival
**PR**	Partial Response
**RRMM**	Relapsed/Refractory Multiple Myeloma
**RT**	Radiation Therapy
